# Supplementary material for: Increased Risk of Tourette Syndrome with Leukotriene Modifier Use in Children with Allergic Diseases and Asthma: A Nationwide Population-Based Study
Source: Children (Basel). 2022 Oct 22;9(11):1607. doi: 10.3390/children9111607 (PMC9688072; doi:10.3390/children9111607)
Supplement: Supplementary file 1 [file children-09-01607-s001.zip › children-1879853-supplementary.pdf]

Table S1: Crude hazard ratio and adjusted hazard ratio for Tourette syndrome among children and adolescence groups between genders

Outcome	Study Group 1	Study Group 2	Comparison Group
	Patients with asthma or allergic rhinitis or atopic dermatitis		
	With receiving LTRA	Without receiving LTRA	People without asthma or allergic rhinitis or atopic dermatitis and without receiving LTRA
**Children male (5-12 y/o)**	**N = 10,037**	**N = 10,037**	**N = 7,907**
Tourette syndrome (n (%))	410 (4.08)	315 (3.14)	65 (0.82)
Crude HR (95%CI)	1.354 (1.169, 1.568)***<br>$p < 0.0001$	1	
Adjusted HR (95%CI)	1.214 (1.036, 1.423)*<br>$p = 0.0167$	1	
Crude HR (95%CI)		3.684 (2.820, 4.812)***<br>$p < 0.0001$	1
Adjusted HR (95%CI)		3.366 (2.571, 4.405)***<br>$p < 0.0001$	1
**Children female (5-12 y/o)**	**N= 7,001**	**N= 7,001**	**N=6,240**
Tourette syndrome (n (%))	132 (1.89)	75 (1.07)	24 (0.38)
Crude HR (95%CI)	1.861 (1.401, 2.472)***<br>$p < 0.0001$	1	
Adjusted HR (95%CI)	1.587 (1.172, 2.150)**<br>$p = 0.0029$	1	
Crude HR (95%CI)		2.601 (1.641, 4.123)***<br>$p < 0.0001$	1
Adjusted HR (95%CI)		2.508 (1.576, 3.989)***<br>$p = 0.0001$	1

Adolescence male (12-18 y/o)	N = 1,589	N = 1,589	N =1,589
Tourette syndrome (n (%))	15 (0.94)	10 (0.63)	5 (0.31)
Crude HR (95%CI)	2.001 (0.893, 4.487) *p* = 0.0921	1	
Adjusted HR (95%CI)	1.543 (0.617, 3.860) *p* = 0.3537	1	
Crude HR (95%CI)		1.536 (0.523, 4.517) *p* = 0.4351	1
Adjusted HR (95%CI)		1.506 (0.504, 4.502) *p* = 0.4636	1
**Adolescence female (12-18 y/o)**	**N = 1,333**	**N = 1,333**	**N =1,333**
Tourette syndrome (n (%))	8 (0.60)	5 (0.38)	0
Crude HR (95%CI)	2.334 (0.757, 7.193) *p* = 0.1399	1	
Adjusted HR (95%CI)	1.918 (0.553, 6.646) *p* = 0.3045	1	
Crude HR (95%CI)		X	1
Adjusted HR (95%CI)		X	1

Hazard ratio were adjusted for age, gender, urbanization, ADHD (attention-deficit hyperactivity disorder), anxiety disorder, depression, epilepsy, OCD (obsessive-compulsive disorder), sleep disorder, intellectual disability, autism, conduct disorder, learning disorder and ICS (inhaled corticosteroids)/ LABA (long-acting beta-agonists); LTRA: leukotriene receptor antagonists; CI, confidence interval; HR, hazard ratio; Study Group1: LTRA users; Study Group 2: LTRA non-users; ***: $p < 0.001$, **: $p < 0.001$, *: $p < 0.05$.
